# Cardiopulmonary Bypass and AKI: AKI Is Bad, So Let's Get Beyond the Diagnosis

**DOI:** 10.3389/fped.2019.00492

**Published:** 2019-11-26

**Authors:** Catherine D. Krawczeski

**Affiliations:** Pediatric Cardiology, Nationwide Children's Hospital, The Ohio State University College of Medicine, Columbus, OH, United States

**Keywords:** acute kidney injury, cardiopulmonary bypass, pediatric, heart, kidney

## Abstract

It is now well-established that AKI is a serious and common complication following cardiopulmonary bypass (CPB) in both children and adults, adverse outcomes may occur in the short term as well as long term, with higher incidence of chronic kidney disease, increased healthcare utilization and higher frequency of cardiovascular events in patients who develop post-CPB AKI. Despite the advances in our understanding of the pathogenesis of the disease and the improvement in diagnostic tools, our therapeutic options have remained suboptimal. There are multiple challenges in designing a clinical therapeutic AKI trial, including a multi-factorial etiology, difficulties with accurate diagnosis of AKI, achievement of adequate study power, and determination of appropriate outcomes. We are often left with “supportive” care. Studies have shown some benefit to AKI bundles, but adherence to bundle guidelines may be suboptimal. Current best practices should include maintenance of adequate renal perfusion pressure and avoidance of fluid overload, with consideration of early renal replacement therapy. Finally, multi-center trials of AKI therapies are crucial to finding treatment for this devastating complication of CPB.

## Introduction

It is now well-established that AKI is a serious and common complication following cardiopulmonary bypass (CPB) in both children and adults, leading to worse outcomes and higher mortality (1–3). While variable, most pediatric studies report an incidence of 30–50%, with higher rates in neonates, more complex surgeries, and longer cardiopulmonary bypass times (2–5). Perioperative AKI is not only associated with higher in-hospital mortality but also longer need for mechanical ventilation, longer intensive care and hospital lengths of stay, and worse ventricular function on discharge (2, 4–6). Adverse outcomes may also occur in the long term, with higher incidence of chronic kidney disease, increased healthcare utilization and higher frequency of cardiovascular events in patients who develop post-CPB AKI (7–10). Despite the advances in our understanding of the pathogenesis of the disease and the improvement in diagnostic tools, our therapeutic options for cardiac surgery-associated AKI have remained suboptimal. This manuscript will review the current status of clinical trials, the inherent problems with these trials, the use of supportive care bundles and potential promising therapies for AKI.

Before launching into potential AKI therapies, it is crucial to understand the pathogenesis of AKI after CPB, as doing so may allow the development of targeted treatment. The mechanism of injury after CPB is multi-factorial, with variable contributions from each process affecting the ultimate AKI phenotype. Thus, AKI after CPB is not homogenous and treatment may not be one-size-fits-all. In addition to ischemia and reperfusion, CPB is associated with alterations in hemodynamics, vasoconstriction and loss of pulsatile blood flow. These factors may lead imbalances between oxygen supply and demand, leading to cellular injury. Activation of the systemic inflammatory response further potentiates cell damage via oxidative stress injury and coagulopathy. CPB also exposes blood cells to non-physiologic surfaces and shear forces, leading to cell lysis and release of plasma free hemoglobin into the circulation. This and other microemboli contribute to further tubular damage. On a cellular level, ischemic injury leads to profound ATP depletion and nitric oxide generation. A number of oxidative and cell death mechanisms are induced, including activation of caspase, increase in intracellular calcium, and generation of reactive oxygen molecules. During the extension phase, these pathways progress, resulting in apoptosis, cell membrane alterations, cytoskeletal degradation, and oxidant injury (11).

## Therapeutic Targets

A number of therapies with specific targets have been studied in clinical trials, including vasodilators, iron chelators, anti-inflammatory agents, anti-apoptotic agents, and diuretics. While some studies have shown modest improvement, no agent has seen universal success. Park et al. reviewed over 500 therapeutic AKI studies from 1950 to 2008 and noted several issues with these clinical trials (12). First and foremost is that most were single center trials and were underpowered to evaluate clinical outcomes. Secondly, the primary outcome in the majority of studies was laboratory based, rather than clinical, such as mortality or need for RRT. Lastly, the definition of AKI was highly variable over the studies, making comparisons difficult. While the latter has been addressed to some extent with the development of consistent AKI definitions such as KDIGO (13), clinical trials remain plagued by insufficient power. Faubel et al. noted this in a 2012 review of ongoing clinical trials in AKI (14). As an example of the difficulty in achieving adequate power, the authors reference the TRIBE-AKI multi-center AKI study (15), which had a 5% incidence of severe AKI, using AKIN (16) serum creatinine criteria. Using these data, to have an α = 0.05, power of 0.9 and 30% effectiveness, almost 3,800 patients per arm would be needed in a randomized controlled study. Even with an enriched sample of high-risk patients, with a 20% incidence of AKI, over 800 patients would be needed in each arm of a study. While this sample number is difficult even in adult studies, it becomes nearly impossible in pediatric studies. Further, as with the earlier review by Park, the authors emphasize that, rather than laboratory based criteria, AKI trials need a clinically important endpoint, such as requirement for renal replacement therapy, quality of life measures, development of chronic kidney disease or mortality, and propose a national AKI clinical trials network to continue this important work.

As therapeutic options for AKI remain limited, the mantra around AKI management is “supportive care.” This includes limiting fluid intake to avoid fluid overload, maintaining adequate cardiac output and blood pressure, avoiding high central venous pressure, augmenting fluid loss, avoiding nephrotoxins, and waiting for kidney recovery. While much of this seems intuitive, adherence to these recommendations often proves difficult. As an example, a 3 kg infant receiving total fluids at “2/3 maintenance” immediately after surgery has a fluid allotment of just 8 ml per hour. This amount would need to include any inotropic or vasoactive medications, antibiotics, pressure monitoring lines, sedation and analgesia, as well as dextrose. As one can easily imagine, fluid from these alone may be more than “maintenance,” even without blood products or true nutrition. Fluid accumulation may be further exacerbated by oliguria, which is common after cardiac surgery. Nephrotoxin exposure is also common in the cardiac intensive care unit (ICU). In a retrospective study of cardiac surgical patients using the NINJA collaboration definition (17), Uber et al. found that 85% of cardiac ICU patients received at least 1 nephrotoxin and 21% received ≥3 nephrotoxins, demonstrating suboptimal adherence this recommendation as well (18). The most common nephrotoxin that was administered was non-steroidal anti-inflammatory agents (ketorolac and/or ibuprofen), which are used widely in post-operative pediatric cardiac patients and have been associated with subclinical kidney injury even in patients without AKI (19).

### Impact of Hemodynamics

The role of blood pressure in avoiding or treating AKI is less clear. Significant hypotension during non-cardiac surgery has been associated with AKI, with higher rates of AKI in patients with mean arterial pressure <55 mmHg, even for short durations (20). While overt hypotension is clearly undesirable, “ideal” blood pressure to prevent AKI and the impact of minor hypotension are unknown. Studies of both healthy adults undergoing hip arthroplasty under controlled hypotension (21) and critically ill patients with sepsis (22) found no association between systemic blood pressure and AKI. However, the latter study demonstrated a significant association with central venous pressure, indicating that it is likely that adequate renal perfusion pressure, rather that systemic blood pressure alone, is key. Similar findings have been seen in pediatric studies of patients undergoing Fontan palliation, a physiology that is often associated with high CVP. A study by Algaze et al. found a significant association between higher post-operative CVP and the development of AKI in 158 patients after Fontan surgery (23). This finding has also been demonstrated in adult studies of patients with advanced decompensated heart failure. Mullens et al. found that worsening renal function (WRF) was associated with higher CVP on admission with a stepwise worsening in function with increasing CVP (24). Systolic blood pressure was not significantly different in patients with or without WRF and the development of WRF uncommon if CVP was <8 mmHg on admission. It should noted, however, that the elevated CVP and AKI are inter-related, since fluid overload from AKI may lead to increases in CVP, so determining cause and effect may be challenging at times.

### Diuretics

Diuretic use to augment fluid loss, while used ubiquitously in the cardiac ICU, has not consistently been shown to improve outcomes and indeed, may be detrimental in some circumstances (25, 26). Lassnigg et al. noted in a single center study that continuous furosemide had no clinical benefit and was associated with increased incidence of AKI in 126 patients after cardiac surgery (27). In a meta-analysis of nine prospective randomized controlled trials, Ho and Sheridan found that furosemide was not associated with any clinical benefit, including decreased mortality, need for RRT, time to recovery of renal function or percent of patients with oliguria (28). One recent study demonstrated lower post-operative serum creatinine in patients receiving intra-operative and early post-operative furosemide but no difference in AKI incidence between study groups (29). Additionally, almost all diuretics must reach the tubular lumen by glomerular filtration or proximal tubular secretion to exert their action (30). If AKI causes a decrease in glomerular filtration, diuretic delivery is impeded and these medications are less effective.

With the thought that the detrimental effect of diuretics on outcomes may be related to intravascular depletion, investigators have looked at the impact of combined administration of diuretics and matched hydration which has been shown to have potential benefit in contrast-associated AKI (31). The impact of this management strategy in CPB-associated AKI is unknown, with an initial, small study of 10 at-risk adult patients demonstrating no AKI after CPB procedures (32). Further study of this management is warranted.

### Supportive Care Bundles

The prevention of AKI using a supportive care “bundle” was evaluated in the PrevAKI trial (33). In this single center randomized controlled trial, 276 patients were randomized to a “KDIGO bundle” consisting of optimization of volume and hemodynamics, avoidance of nephrotoxins and prevention of hyperglycemia, or to standard care. The primary outcome was rate of AKI using KDIGO guidelines. The intervention group was found to have significantly less AKI and severe AKI. The intervention group received more dobutamine resulting in higher mean arterial pressure (and thus higher renal perfusion pressure), had less hyperglycemia, and received less angiotensin-converting enzyme inhibitors or angiotensin II receptor blockers. There was no difference in need for renal replacement therapy or major adverse events between groups.

### Fenoldopam

While supportive care is crucial, the development of AKI therapies remains a priority. Several therapies have shown some benefit in select populations and likely warrant further investigation in larger trials. Fenoldopam, a selective dopamine-1 receptor agonist, increases renal plasma flow, decreases renal vascular resistance, and inhibits tubular resorption of sodium. It was first evaluated in neonates after cardiac surgery who had insufficient response to conventional diuretics (34). In this single center study, neonates receiving fenoldopam had a significant increase in urine output. Ricci et al., in a randomized controlled trial of high-dose fenoldopam in children after cardiac surgery, found a decrease in post-operative neutrophil gelatinase-associated lipocalin and cystatin C levels and a trend toward less AKI, sooner extubation and shorter LOS in the treatment group (35). In a meta-analysis of 23 studies, fenoldopam treated patients had a significantly lower incidence of AKI, with an OR of 0.46 [0.27–0.79] but no difference was seen in mortality, rate of RRT or other clinical outcomes (36). This meta-analysis included both cardiac and non-cardiac surgical patients with differing durations of therapy so while promising, further multi-center studies are warranted.

### Aminophylline

Aminophylline is a methylxanthine non-selective adenosine receptor antagonist and has been shown to decrease adenosine mediated vasoconstriction, inhibit phosphodiesterase, and increase urine output. One of the earliest single center double-blinded placebo controlled clinical trials in pediatric patients after CPB did not show significant differences in AKI incidence or difference in secondary clinical outcomes between treatment and control groups (37). Other studies however, have found variable effects, with several reporting improved clinical outcomes and lower incidence of AKI (38, 39).

### Dexmedetomidine

Dexmedetomidine, an alpha-2 agonist, is used primarily for sedation. It is noted to have sympatholytic, cytoprotective and anti-inflammatory properties and has recently been evaluated in AKI studies. Kwiatkowski et al. noted significantly less AKI by KDIGO [adjusted OR 0.43 (0.27–0.98)] following congenital heart surgery but did not find differences in clinical outcomes (40). A prospective randomized trial in pediatric CPB patients also found a significant decrease in AKI in the treated group but also failed to show a difference in clinical outcomes (41).

### Fluid Overload

The avoidance of fluid overload (FO) is perhaps the most important target for AKI intervention, as FO has independently been associated with worse outcomes including mortality in AKI (42). Early RRT has been associated with improvement in clinical outcome in both adult and pediatric patients and earlier institution of RRT has been associated with better survival (43). The concept of early, or “prophylactic” peritoneal dialysis has been around for decades (44–46) and its use is becoming more common (47). Several centers routinely place dialysis catheters intra-operatively at the time of cardiac surgery in high-risk patients, using a trans-diaphragm approach. Catheters are placed to passive drainage and if early signs of AKI occur, such as oliguria, peritoneal dialysis is begun with goal to avoid fluid overload, not necessarily to achieve net negative fluid balance. After a retrospective study of PD catheter placement after CPB in infants demonstrated improved clinical outcomes, including time to extubation and degree of FO (48), Kwiatkowski et al. embarked on a prospective randomized trial of PD vs. standard regimen of furosemide (47). This study found that PD patients were 3 times less likely to have 10% FO, achieved negative fluid balance one shift sooner, and had improved clinical outcomes including less likelihood of prolonged ventilation, or prolonged ICU stay. Importantly, PD patients had no adverse events associated with catheter placement or use. Thus, it may be that prevention of FO with consideration of early RRT or PD is our current best “treatment.”

## Conclusion

While significant work has been done in the study of AKI and potential treatment, a single therapeutic strategy remains elusive. There are multiple challenges in designing a clinical therapeutic AKI trial, including a multi-factorial etiology, difficulties with accurate diagnosis of AKI, achievement of adequate study power, and determination of appropriate outcomes. We are often left with “supportive” care. Studies have shown some benefit to AKI bundles, but adherence to bundle guidelines may be suboptimal. Current best practices should include maintenance of adequate renal perfusion pressure and avoidance of fluid overload, with consideration of early renal replacement therapy. Finally, multi-center trials of AKI therapies are crucial to finding treatment for this devastating complication of CPB.

## Author Contributions

The author confirms being the sole contributor of this work and has approved it for publication.

### Conflict of Interest

The author declares that the research was conducted in the absence of any commercial or financial relationships that could be construed as a potential conflict of interest.
